# Dual-energy computed tomography in supporting prostatic artery embolization for benign prostatic hyperplasia

**DOI:** 10.7150/ijms.96319

**Published:** 2024-06-17

**Authors:** Le Thanh Dung, Nguyen Duy Hung, Le Quy Thien, Dang Khanh Huyen, Le Nguyen Vu, Duong Duc Hung, Nguyen Minh Duc

**Affiliations:** 1 Department of Radiology, VNU University of Medicine and Pharmacy, Vietnam National University, Hanoi, Vietnam.; 2 Department of Radiology, Viet Duc Hospital, Hanoi, Vietnam.; 3 Department of Radiology, Hanoi Medical University, Hanoi, Vietnam.; 4 Department of Radiology, Tam Anh Hospital, Hanoi, Vietnam.; 5 Organ transplantation center, Viet Duc hospital, Hanoi, Vietnam.; 6 Cardiovascular and thoracic surgery center, Viet Duc hospital, Hanoi, Vietnam.; 7 Department of Radiology, Pham Ngoc Thach University of Medicine, Ho Chi Minh City, Vietnam.

**Keywords:** dual-energy computed tomography, embolization, prostatic artery, benign prostatic hyperplasia

## Abstract

**Objective:** Our study aims to evaluate the value of 256-slice dual-energy computed tomography (DECT) in supporting prostatic artery embolization (PAE) under digital subtraction angiography (DSA) for benign prostatic hyperplasia (BPH).

**Methods:** The study was conducted on 88 patients who underwent PAE to treat BPH from January 2022 to November 2023. Of these, 38 patients who had PAE without DECT were placed in group 1, while the other 50 patients with pre-interventional DECT were assigned to group 2. The results of DECT imaging of the prostate artery (PA) were compared with the results of DSA imaging. Test for statistically significant differences between the variables of the two research groups using the T - student test and Mann-Whitney test algorithms with p < 0.05 corresponding to a 95% confidence interval. The data were analyzed according to medical statistical methods using SPSS 20.0 software.

**Results:** DECT can detect the PA origin in 96.1% of cases, identify atherosclerosis at the root of the artery with a sensitivity of 66.7% and a specificity of 89.5%, and present anastomosis with a sensitivity of 72.7% and a specificity of 72.2%. There is no statistically significant difference in PA diameter on DECT compared to DSA with 95% confidence. Group 2 used DECT for 3D rendering of the PA before PAE had procedure time reduced by 25.8%, fluoroscopy time reduced by 23.2%, dose-area product (DAP) reduced by 25.6%, contrast medium volume reduced by 33.1% compared to group 1 not using DECT, statistically significant with 95% confidence.

**Conclusion:** DECT is a valuable method for planning before PAE to treat BPH. 3D rendering DECT of PA provides anatomical information that minimizes procedure time, fluoroscopy time, dose-area product, and contrast medium volume.

## Introduction

Prostatic hyperplasia is a common benign disease of the urinary system, often appearing in older men, causing symptoms of lower urinary tract obstruction. The incidence of BPH increases with age and has been increasing in Vietnam and around the world in recent years 1.

Among a group of several currently developed BPH treatment methods, the tendency to use the PAE method is increasingly popular. PAE was first reported in 2000 for the treatment of lower urinary tract symptoms caused by BPH and has been increasingly developed and widely used 2. The advantages of PAE are minimal invasiveness, quick recovery time, and low risk of complications. It is suitable for patients taking anticoagulants and patients who are contraindicated for general anesthesia or spinal anesthesia 3. However, there is a major technical challenge of the PAE, which is the identification and placement of the arterial catheter 1, 4, 5, 6. Due to the complexity of PA anatomy, there is a large number of PA variant anatomy between patients and between two sides of the pelvis in the same patient 7, 8. Small PA diameter, inconsistent number of branches and origins of PA, complex anatomical variations, and frequent atherosclerosis in the elderly lead to difficulties during intervention 6. Therefore, accurately determining the anatomical characteristics of the PA before intervention is essential to reduce intervention time and radiation dose.

Dual-energy computed tomography (DECT) is a step forward in imaging diagnosis technology, especially valuable in assessing small-sized artery disease. In addition, the ability to remove bone during rendering is a feature that provides optimal multidimensional imaging in vascular assessment 9, 10. Current imaging techniques, such as MRA (magnetic resonance angiography), DSA, and Conebeam CT, CTA (computed tomographic angiography) have been used for identifying the prostate arterial anatomy prior to PAE or during PAE 4, 11. However, each of these techniques has its own limitations during this process. Solitary conventional DSA is restricted to identifying the PAs due to the superimposition of viscera in the lower pelvis and perineum. CTA or MRA has limited resolution to detect small PAs. Meanwhile, DECT with the ability to remove bone during rendering has been proven to be much better than CTA or MRA in detecting and evaluating small arteries 9, 10. In the medical literature, there are only a few studies on diagnostic imaging techniques with PA rendering before PAE, mainly evaluating conventional CTA, cone-beam CT, and MRA 4, 6. According to our knowledge, there was previously no study using DECT to evaluate PAs before PAE. Therefore, the purpose of our study is to evaluate the value of 256-slice DECT in PA rendering to support the PAE.

## Methods

The study was conducted on 88 patients who received PAE to treat BPH at the Department of Diagnostic Imaging of Viet Duc Hospital from January 2022 to November 2023. The patients in the study were divided into 2 groups: 38 patients who had PAE without DECT were placed in group 1, and the other 50 patients with pre-interventional DECT were assigned to group 2. The research was conducted under consent, ensuring the rights and health of patients. Medical records and images are kept confidential and used solely for research purposes. The data in the study is ensured to be accurate.

Scan parameters of DECT Revolution HD (GE) machine in our research: fast kilovoltage switching between 80 and 140kVp, tube current of 600 mA, rotation speed of 0.5s, pitch of 1.375:1, slice thickness of 5mm, reconstruction slice thickness of 0.625 mm, contrast injection speed of 5ml/s, contrast dose of 1.5 ml/kg body weight, DLIR High reconstruction algorithm, AW 4.7 processing station. The scanning delay for the mobile phase was determined using automatic scan-triggering software (GE Healthcare). Scanning was performed in simultaneous spectral imaging mode with fast kilovoltage switching between 80 and 140kVp on adjacent views in one rotation. The image data set reconstructed from the single spectrum acquisition consists of one set of polychromatic images (corresponding to conventional 120 kVp images) and one set of monochromatic images read at low KeV (40 - 50KeV) is considered optimal for evaluating small arteries.

The 256-slice dual-energy computed tomography is the 4th generation of GE machines that was licensed for use by the Food and Drug Administration (FDA) in 2014. It offers quick scan times, high image quality, and an 82% lower radiation dose per scan compared to previous generations of scanners^12^. In addition, 256-slice CT had a shorter scan time compared to 64-slice CT (4.4 ± 0.6 seconds vs. 5.0 ± 0.7 seconds, p < 0.001). Based on analysis of patient data, the accuracy rate of diagnosing vascular diseases and the presence of severe calcification of 256-slice CT were much higher than 64-slice CT 13. Single-energy imaging at lower peak kilovoltage (<70 keV) shows higher contrast in iodine-containing tissues because of the higher photoelectric effect due to the near-threshold energy absorption of iodine (33KeV), which increases the vascular image quality of DECT. In addition, the ability to remove bone during rendering is a feature that provides optimal multidimensional images in vascular assessment.

The images were evaluated and analyzed by radiologists with over 5 years of experience in PAE. Test for statistically significant differences between the variables of the two research groups using the independent sample T-test and Mann-Whitney test algorithms with p < 0.05 corresponding to a 95% confidence interval. The data were analyzed according to medical statistical methods using SPSS 20.0 software.

## Results

Our study was conducted on 88 patients (mean age 71.2 ± 7.7) who underwent PAE to treat BPH from January 2022 to November 2023, divided into two groups. 38 patients who underwent PAE without DECT were classified into group 1, and group 2 contains the remaining 50 patients with pre-interventional DECT. Furthermore, 02 patients had PAE on only one side, and 86 patients had PAE on both sides.

### General characteristics

The general epidemiological, clinical, and laboratory characteristics of the two study groups are shown in **Table 1**. The study results showed that there were no statistically significant differences between age, IPSS, Qol scores, prostate volume, and PSA level between the two study groups with 95% confidence (p > 0.05).

### The value of 256-slice dual-energy computed tomography in visualizing the PA before PAE

We studied 50 patients of group 2 (corresponding to 100 pelvic sides) with 104 PAs confirmed by DSA. These patients underwent DECT before PAE, helping to compare the value of DECT and DSA in assessing PA before intervention.

In 50 patients who underwent DECT before PAE to treat BPH, DECT was able to detect the root of the PA of 96.1% (100 out of 104).

In detecting atherosclerosis of the PA, DECT had a sensitivity of 66.7% and a specificity of 89.5%.

In detecting anastomoses of PA, DECT was capable of detecting 32 anastomoses, including 20 false positives and 18 false negatives, with a sensitivity of 72.7% and a specificity of 72.2% (compared to DSA detecting 48 anastomoses). In our study, the most common anastomosis was with contralateral PA (58.3%), followed by the penis (18.75%), rectum (12.5%), and bladder (10.4%).

There was no significant difference between the average diameter of the PA between the two study groups with 95% confidence (p = 0.125 > 0.05).

### Evaluating the value of DECT in supporting the PAE

The group 2 using 3D-rendering DECT to support selective guidance of the PA had procedure time reduced by 25.8%, fluoroscopy time reduced by 23.2%, dose-area product dose reduced by 25.6%, and contrast medium volume reduced by 33.1%. All changes are statistically significant with 95% confidence (p < 0.05).

## Discussion

Prostatic artery embolization (PAE) is a safe and effective treatment for BPH. The main technical challenge of this intervention is to identify and catheterize the PAs, especially the localization of the PAs, morphology, anatomical variations, and atherosclerosis arteries, leading to prolonged procedure times and receiving higher radiation doses 5, 6. Therefore, predicting the origin, diameter, tortuosity, and atherosclerotic occlusion of the PA, and the number of PAs can help radiologists plan treatment and exclude patients who are not suitable for PAE due to arterial anatomy. It helps reduce procedure time and dose radiation, avoiding the risk of unintentional embolism in surrounding organs and nontarget embolization (bladder, rectum, or penis). Currently, the PA is determined mainly by performing DSA and accompanied by Conebeam CT, CTA or MRA, etc. Research by author Tiago Bilhim 14 had shown an excellent correlation between CTA and DSA imaging in evaluating PAs prior to PAE. CTA provided precise details of PA characteristics, aiding in treatment planning and ruling out patients with unsuitable arterial anatomy that could impact the procedure's success 14. The effectiveness was evident when PAs arose from the external iliac artery, such as the accessory obturator artery. Preprocedural pelvic CTA can provide helpful information about pelvic vessels and PA anatomy to reduce the procedure time and radiation exposure and increase the technical success rate. Disadvantages of CTA include increasing the burden of time, contrast medium volume, and radiation on patients. In addition, CTA lacks sensitivity for identifying some small PAs. The study by author Kim *et al.* 4 evaluated the ability and demonstrated the role of MRA in determining the origin of the PA before the PAE on a small sample and did not include a control group. In the medical literature, there is currently no study using DECT scans to evaluate the PA before intervention for BPH.

The average age of patients in the two study groups was 67.6 ± 13.3 years and 62.4 ± 10.3 years, respectively (age range from 43 to 88 years old), with no statistically significant difference with 95% confidence (p > 0.05) (**Table 1**). The age of the patients with PAE in our study was similar to other studies, usually in the middle-aged and elderly groups because the research subjects were often patients with BPH. Research by author Tiago Bilhim *et al.* 15 was conducted on 75 patients with an average age of 66 (from 50 to 80 years old). In the research by author Nguyen Xuan Hien *et al.* 16, there were 58 patients ranging in age from 53 to 91. This age group is often accompanied by other chronic diseases such as hypertension, diabetes, and heart failure. Therefore, choosing a less invasive intervention method is reasonable to avoid possible complications in surgery.

Our study results showed no statistically significant difference between IPSS scores, Qol scores, and serum PSA levels between the two study groups with 95% confidence (p > 0.05). The IPSS score evaluates lower urinary tract symptoms, including 7 questions with two groups of obstructive and irritant symptoms 17. In contrast, the Qol score is a score to assess quality of life. IPSS scores and Qol scores of patients in the two study groups were respectively 24.4 ± 4.6 and 3.5 ± 2.3; 23.6 ± 3.5 and 3.1 ± 2.2, corresponding to severe obstruction (IPSS score from 20 - 35 points) and moderate quality of life, consistent with the criteria for indications for PAE intervention to treat BPH. Total PSA concentrations of patients in the two study groups were 7.5 ± 3.5 and 9.4 ± 3.7, respectively, and a slight increase in the range of 4 - 10 ng/mL is appropriate for the BPH (**Table 1**).

The average prostate volume of the two groups in our study was 74.3 ± 15.6 ml and 89.4 ± 18.5 ml, respectively, with no statistically significant difference with 95% confidence (p > 0.05). Among them, the largest prostate volume was 210 ml. The large prostate volume is not a difficulty for PAE. The study by Wang *et al.* 11 in 2015 showed that embolization is a safe and effective method for cases with prostate enlargement over 80 grams. Meanwhile, most surgeons believe that a weight of average above 80 grams is a challenge with laparoscopic surgery, even with open surgery^18^. Thus, embolization is a suitable method for patients with large prostate volumes who cannot have surgery or have failed surgery.

Evaluation of the group 2 in the study included 50 patients who received DECT scans before embolization to treat BPH. The results showed that the ability to detect PA on DECT was almost equivalent to DSA with a detection rate of 96.15%, no false positives, false negative rate of 3.85%. The study of Maclean *et al.* 18 evaluated the value of CTA in identifying the PA and the anastomoses, showing that the detection rate of the PA was 97.3%, equivalent to the results of our study. Author Kim *et al.* 4 evaluated the effectiveness of MRA in determining the origin of the PA before the intervention, the results showed that 26/34 PAs were identified (accounting for 76.5%) in 17 patients. In addition, in our study, the PA originating from the common trunk with superior vesical artery accounted for the highest proportion (33.2%), and the PA originating from the obturator artery was the second most common. Research by author Nguyen Xuan Hien^16^ showed similar results, the most common location was from the common trunk with superior vesical artery (33.1%) and the second most common location was from the internal pudendal artery. On DECT images, determining the origin of the PA is based on the image of the artery's path from the glandular parenchyma towards the origin, due to the ability to construct MIP and 3D images and locate points on planes (**Fig. 2**) (**Fig. 3**). In addition, less common locations of origin of the PAs are the superior gluteal artery (SGA), inferior mesenteric artery (IMA), middle rectal artery, accessory internal pudendal artery (IPA), accessory obturator artery (OA), or others. In our study, the rate of rare locations (type V) was 6%, equivalent to FC Carnervale's classification 6. In cases where the PA cannot be found when selecting the arterial branches internal iliac, especially when the ipsilateral obturator artery is not observed, then angiography of the external iliac artery should be performed to find the PA separating from the accessory obturator artery (OA) (**Fig. 3**). In this case, the interventionist should not let the embolization material reflux out of the PA because there is a lower limb artery occlusion risk. In addition, the PA variant originating from the accessory pudendal artery is considered the most important variant in the type V variant group because the accessory pudendal artery often has a parallel path and connects with the internal pudendal artery, so it is often very short and prone to reflux when injecting paralysis material. To avoid non-target embolization in the penile area, it is necessary to use a coil or sponge to block this connection 19. Our study encountered 03 cases of PA originating from the accessory pudendal artery with a rate of 0.8%. DECT imaging to evaluate the PA before intervention will be helpful in pre-identifying these rare cases, helping to reduce the time to select the PA. In addition, our study also found that there was asymmetry in the origin of the pelvic artery on both sides of the pelvis in the same patient in 74.3%, similar to the study of author Wang 11. This suggests that each side of the pelvis should be considered independent when selective arterial catheterization and embolization are performed.

Another difficulty in the process of PAE is atherosclerosis. Atherosclerosis makes performing super-selective arteries more difficult and directly affects the results of the embolization technique. In our study, the rate of atherosclerosis observed was 7.7%. DECT can detect atherosclerosis at the base of the artery with a sensitivity of 66.7% and a specificity of 89.5%.

The PA usually has a gradually decreasing diameter along the way, the closer it is to the parenchyma of the gland, the smaller the diameter. Because the PA is small and its path is quite variable, the average diameter of each PA was calculated as the average of the diameter when measured at 3 locations, including the starting location of the PA (proximal point), the location about to branch (far point) and and the location between these two locations. The average diameter of the PA in our study was 1.5 mm, equivalent to the results of Wang *et al.* 11, Nguyen Xuan Hien *et al.* 16, and Tiago Bilhim *et al.* 15. There was no statistically significant difference in PA diameter between the two groups in our study. The ability to detect the PA on DECT was 96.1%, and the diameter of the PA on DECT compared to the actual diameter on DSA was 88.2%. In the study by Wang et al. 11, the group of patients with large prostate volume (≥80ml) had larger prostate diameter than the group with small average prostate volume (<80ml), the difference was statistically significant with 95% confidence. In our study, 04 pelvic arteries could not be evaluated on DECT, with the diameter of these arteries being less than 0.6mm. Based on the pre-intervention DECT scan, we can determine the microcatheter and particle sizes to select and plug the prostate artery accordingly.

Our study has shown that the ability to detect anastomoses on DECT has a sensitivity of 72.7% and a specificity of 72.2%. The study by Maclean et al. evaluated the value of multi-slice CTA in identifying the PAs and anastomoses, showing that the ability to detect the PA was 97.3%, and the ability to detect anastomoses had a sensitivity of 59.0%, a specificity of 94.2% 18. It can be seen that the ability to detect PA and PA diameter compared to DSA imaging in our study and the study of Maclean et al. is equivalent. Kim et al. evaluated the effectiveness of MRA in determining the origin of the PA before PAE and identified 26/34 (76.5%) PAs in 17 patients (34 pelvic sides) 4. The results of detecting the anastomoses showed the limitation of DECT in evaluating the anastomoses compared to the Cone-beam CT angiogram because the anastomoses are often very small and need to be confirmed on DSA or can only be detected by DSA. This limitation is due to image noise as well as the discontinuity of the images of anastomoses on DECT, leading to confusion. DECT performed at least 1 day before PAE intervention allows the intervention to be canceled if the degree of atherosclerosis is too severe to perform embolization. Furthermore, it provides additional time for reviewing images prior to commencing intervention, enabling thorough evaluation through various reconstruction planes and 3D visualization. This aids in pinpointing areas of atherosclerosis, torsion, and offering recommendations for arterial entry.

**Figure 1:**
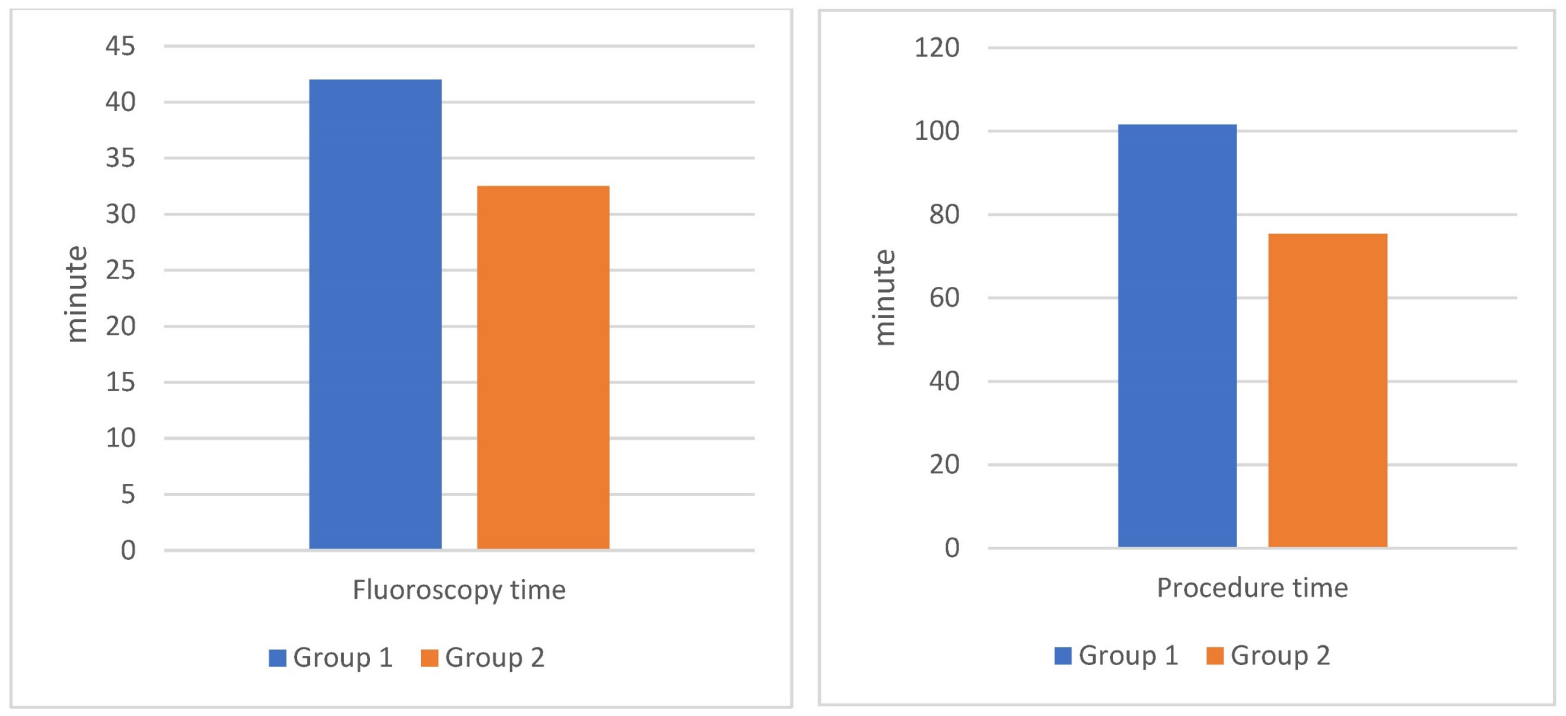
** Comparison of fluoroscopy time and procedure time during PAE between the two groups.** Group 1: patients who underwent PAE without DECT. Group 2: patients who underwent PAE with pre-interventional DECT.

**Figure 2:**
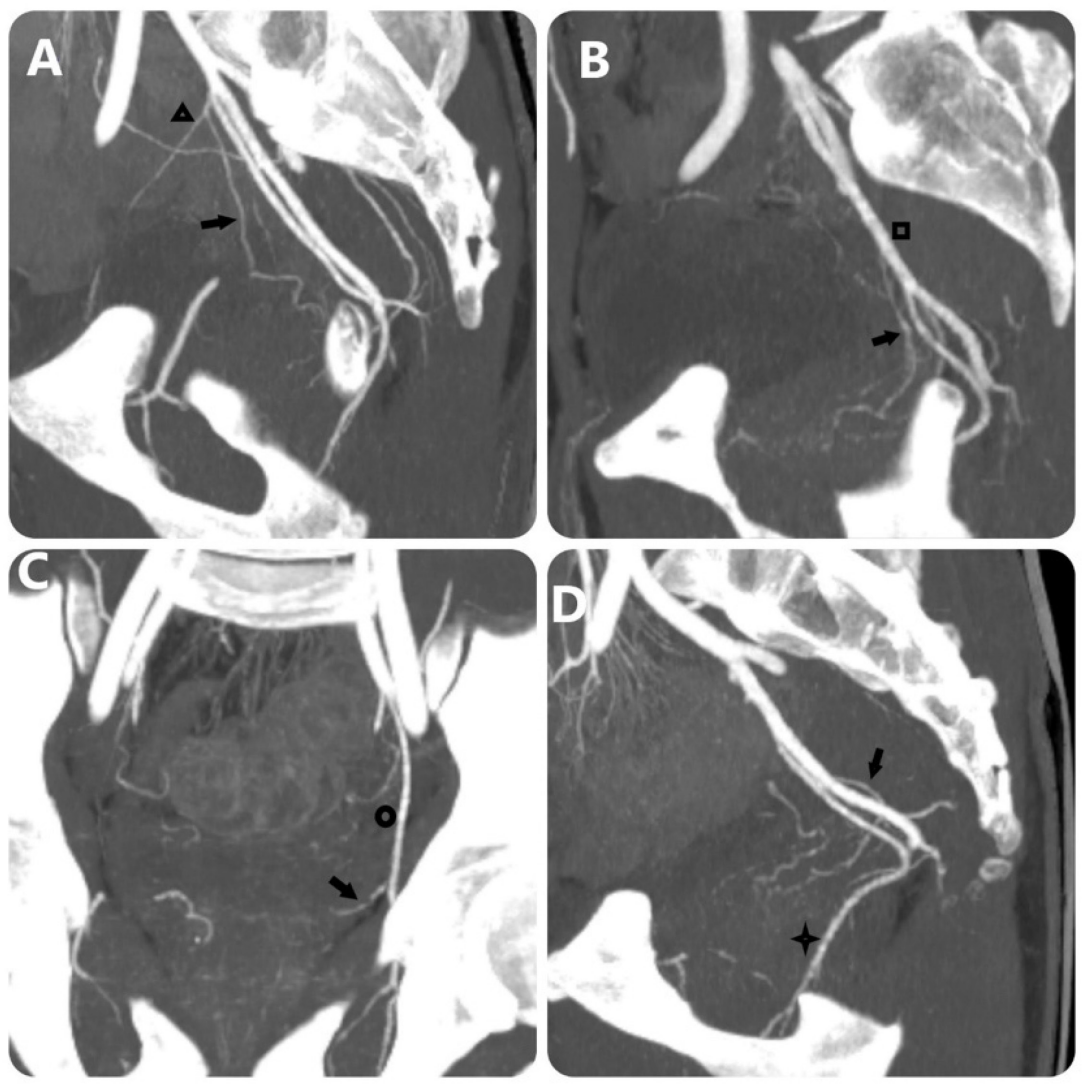
MIP images with 10-mm slice thickness image PA's origin equivalent FC Carnervale's classification: (a) type 1, (b) type 2, (c) type 3, and (d) type 4. (Black arrow: prostate artery, black triangle: superior vesical artery, black square: anterior trunk of internal iliac artery, black circle: obturator artery, black star: internal pudendal artery).

**Figure 3:**
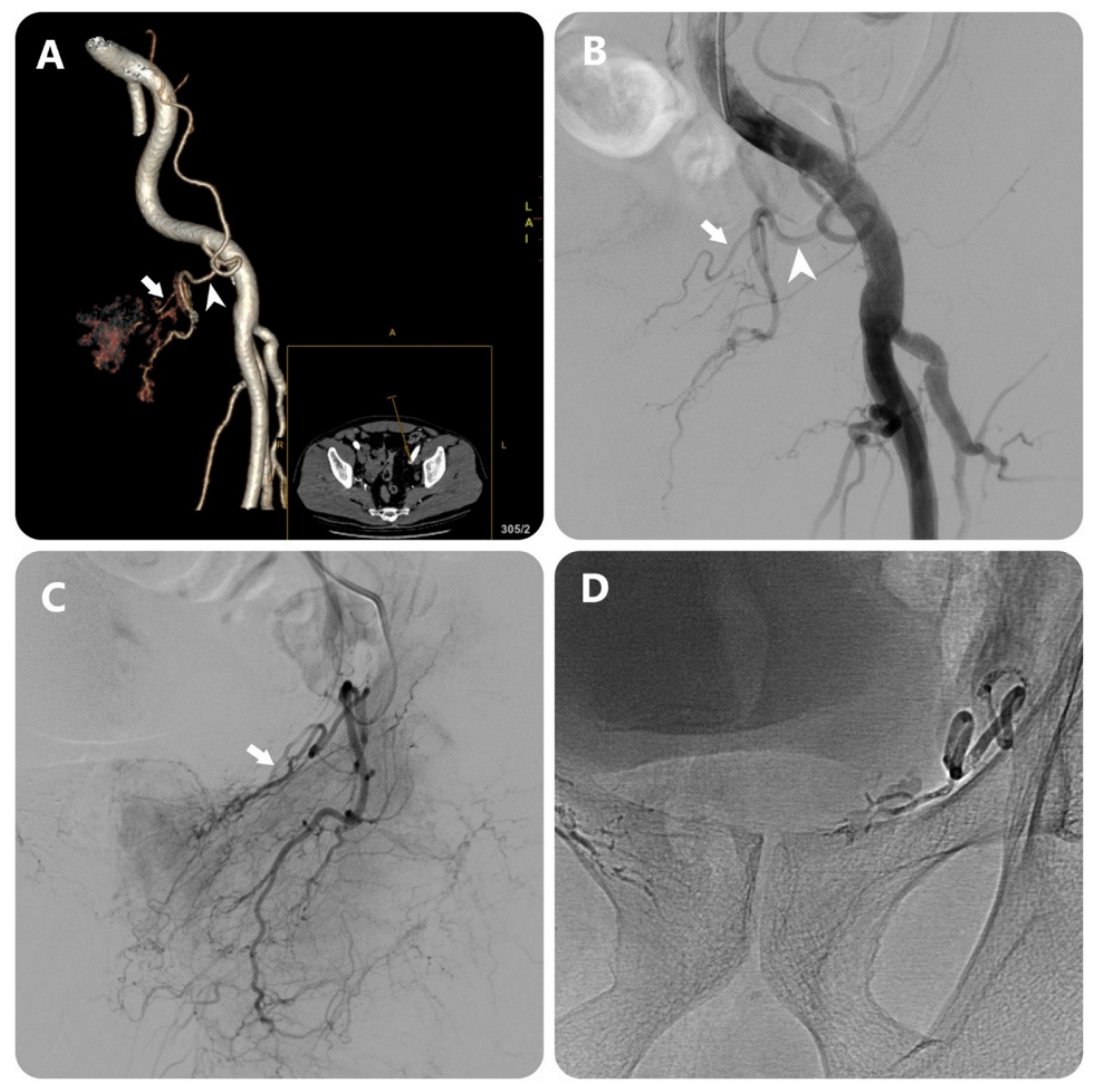
Images in an 80-year-old man with symptomatic BPH (prostate volume 94 mL). (a) 3D image of the left external iliac artery (EIA) and its branches from DECT data in a 15° contralateral anterior oblique direction shows left PA (white arrow) arising from accessory obturator artery (OA)(white arrowhead) (b) EIA angiography performed with injection of 12 mL of contrast medium at a rate of 6 mL/sec (3 frames per second) (c) OA angiography performed with injection of 7 mL of contrast medium at a rate of 3 mL/sec (3 frames per second) (c) Post-embolization single-shot image.

In our study, group 2 showed a 25.8% reduction in procedure time, a 23.2% decrease in fluoroscopy time, a 25.6% reduction in DAP, and a 33.1% decrease in the use of contrast agents compared to group 1 **(Table 3) (Figure 1)**. These findings were statistically significant with 95% confidence. Research by Zhang JL *et al.* using MRA before PAE intervention demonstrated comparable outcomes: procedure time decreased by 33.6%, fluoroscopy time by 51.6%, and DAP by 35.5%^5^. MRA has the advantage of not being exposed to X-rays, but the cost of MRA is higher and the sensitivity and specificity are also lower than DECT. Drew Maclean *et al.* reported that CTA before PAE was more reliable than MRA for predicting arterial anatomy and facilitating intervention^18^. Directly measuring radiation dose with specialized measuring equipment at locations on the body will give more accurate numbers than the number obtained from the post-intervention radiation dose report on the DSA machine. In published studies on PAE, we found no reports with directly measured radiation dose data.

Among the studies mentioned in **Table 4**, our study had the lowest DAP (many times lower than other studies) and the second shortest fluoroscopy time (only lower than the study by Pisco *et al.*). In 2016, Pisco *et al.* 20 published a study on radiation dose results after PAE on 630 patients who underwent CTA before the intervention, to evaluate anatomy and plan intervention. Their report had the shortest fluoroscopy time but the highest radiation dose (DAP) (**Table 4**), showing that fluoroscopy time is only an indirect factor and does not reflect the amount of radiation received. Research by Maclean^19^ reported on the radiation exposure of patients and interventional radiologists with more than 10 years of experience during PAE to treat BPH in 25 patients who did not receive pre-intervention CTA or MRA. The results of this study showed that 7/25 patients needed CT scan at the table during intervention, the average radiation time was 30.9 minutes (15.5-48.3 minutes), the average total dose was 450.7 Gy.cm^2^ (248.3-791.73 Gy.cm^2^ ). The radiation dose after PAE was proven to be greater than other complex radiological interventions such as the technique of creating an intrahepatic portosystemic shunt through the jugular vein, thrombectomy, and treating liver tumors with embolization chemistry 27. Compared with conventional CTA, DECT has an actual radiation exposure level of about 60% calculated according to the dose-length index (DLP) and the cost is not significantly higher. According to research by Ramin Ghasemi Shayan *et al.* 28, DECT scan had a DLP index of 57.7% compared to conventional single-energy shooting.

In addition, reducing the amount of contrast agent needed for PAE intervention in patients who received DECT before intervention compared to patients who did not was also statistically significant with 95% confidence. This could be achieved by reducing the number of times to select and test the PA, due to already knowing the anatomy of the PA as well as the internal iliac artery before intervention, the appropriate angle. In many cases, we did not need to take a test scan, we just needed to create a map of the internal iliac artery and then select the PA. Patients in this group will undergo a DECT scan at least one day before to avoid the simultaneous use of multiple contrast agents on the same day. Our study has several limitations. First, the sample size is small. Second, there is no comparison of the results between DECT and conventional CTA, compared to MRA. Therefore, we hope to conduct future studies comparing the effectiveness of DECT and MRA in the assessment of PA before PAE. This helps confirm the advantages and disadvantages of the two methods and offer suggestions for use prior to intervention.

## Conclusion

Dual-energy computed tomography (DECT) before intervention to evaluate the prostate artery is a valuable method to plan before endovascular intervention to treat benign prostate hyperplasia. DECT has a good ability to detect and evaluate PA and its anastomoses, thereby helping to reduce procedure time, fluoroscopy time, radiation dose, and amount of contrast agent needed.

### Ethical approval

Hanoi Medical University's institutional review board supported this study. This study was conducted according to the ethical standards of the 1964 Declaration of Helsinki and its later amendments.

### Informed consent

Informed consent was obtained.

### Availability of data and material

The datasets generated and/or analysed during the current study are not publicly available due to privacy concerns but are available from the corresponding author on reasonable request.

### Orcid ID

Nguyen Minh Duc: https://orcid.org/0000-0001-5411-1492.

### Author contributions

Study concept and design: Le Thanh Dung, Nguyen Duy Hung, and Nguyen Minh Duc; acquisition of data: Le Thanh Dung, Nguyen Duy Hung, and Nguyen Minh Duc; analysis and interpretation of data: Le Thanh Dung, Nguyen Duy Hung, and Nguyen Minh Duc; drafting of the manuscript: Le Thanh Dung, Nguyen Duy Hung, and Nguyen Minh Duc; critical revision of the manuscript: Le Thanh Dung, Nguyen Duy Hung, and Nguyen Minh Duc; study supervision: Le Thanh Dung and Nguyen Duy Hung confirm the authenticity of all the raw data. All authors read and approved final version of this manuscript.

## Figures and Tables

**Table 1:** General characteristics of the two patient groups

Characteristic	Group 1 (n=38)	Group 2 (n=50)	P Value
**Age**	67.6 ± 13.3	62.4 ± 10.3	0.24*
**IPSS**	24.4 ± 4.6	23.6 ± 3.5	0.561*
**Qol score**	3.5 ± 2.3	3.1 ± 2.2	0.342*
**Prostate volume (ml)**	74.3 ± 15.6	89.4 ± 18.5	0.245*
**PSA level (ng/mL)**	7.5 ± 3.5	9.4 ± 3.7	0.356*

* Mann-Whitney Test

**Table 2:** Comparison of DECT and DSA in determining the origin, atherosclerosis, anastomosis, and diameter of the PA

Characteristic		DECT	DSA	False positive	False negative
Detecting PA origin		100	104	0	4
Atherosclerosis of the PA		8	6	1	3
Anastomosis	Rectum	3	6	1	4
	Penis	7	9	1	3
	Bladder	2	5	5	3
	Contralateral PA	20	28	13	8
Average diameter		1.5±0.4	1.7± 0.3	p = 0.125*	

* Mann-Whitney Test

**Table 3:** Comparison of parameters during PAE between the two groups

Characteristic	Group 1	Group 2	Percentage reduction	P value
**Fluoroscopy time (minute)**	42 ± 12.4	32.5 ± 6.8	23.2	0.014*
**Procedure time (minute)**	101.6 ± 12.4	75.4 ± 12.3	25.8	<0.01**
**Dose-area product (DAP) (mGy.cm2)**	23914.3 ± 5541.5	17796.2 ± 3823.5	25.6	<0.01*
**Contrast medium volume**	167.67 ± 45.2	108 ± 23.3	33.1	0.023*

* Mann-Whitney Test **Independent Samples Test

**Table 4:** Comparison of fluoroscopy time and DAP during PAE

Research, year	CTA scan before intervention	Fluoroscopy time	DAP (Gy.cm2)
Pisco *et al.* 2016 20	Yes	19.5	2415
Grosso *et al.* 2015^21^	Yes	69	-
Wang *et al.* 2017^11^	No	30	-
Li *et al.* 2015^22^	No	36	-
De Assis *et al.* 2015^19^	No	55.4	-
Gao *et al.* 2014^23^	No	33.2	1130.5
Balga *et al.* 2014^24^	No	30.2	559.2
Carnevale *et al.* 2013^25^	No	85.9	-
Our research	DECT	32.5	177.962
